# Advanced Global Prototypical Segmentation Framework for Few-Shot Hyperspectral Image Classification

**DOI:** 10.3390/s24165386

**Published:** 2024-08-21

**Authors:** Kunming Xia, Guowu Yuan, Mengen Xia, Xiaosen Li, Jinkang Gui, Hao Zhou

**Affiliations:** School of Information Science and Engineering, Yunnan University, Kunming 650504, China; xiakunming@stu.ynu.edu.cn (K.X.); gwyuan@ynu.edu.cn (G.Y.); xiamengen@stu.ynu.edu.cn (M.X.); lixiaosen@stu.ynu.edu.cn (X.L.); guijinkang@stu.ynu.edu.cn (J.G.)

**Keywords:** hyperspectral image (HSI) classification, few-shot learning (FSL), contrastive learning (CL), fully convolutional network (FCN)

## Abstract

With the advancement of deep learning, related networks have shown strong performance for Hyperspectral Image (HSI) classification. However, these methods face two main challenges in HSI classification: (1) the inability to capture global information of HSI due to the restriction of patch input and (2) insufficient utilization of information from limited labeled samples. To overcome these challenges, we propose an Advanced Global Prototypical Segmentation (AGPS) framework. Within the AGPS framework, we design a patch-free feature extractor segmentation network (SegNet) based on a fully convolutional network (FCN), which processes the entire HSI to capture global information. To enrich the global information extracted by SegNet, we propose a Fusion of Lateral Connection (FLC) structure that fuses the low-level detailed features of the encoder output with the high-level features of the decoder output. Additionally, we propose an Atrous Spatial Pyramid Pooling-Position Attention (ASPP-PA) module to capture multi-scale spatial positional information. Finally, to explore more valuable information from limited labeled samples, we propose an advanced global prototypical representation learning strategy. Building upon the dual constraints of the global prototypical representation learning strategy, we introduce supervised contrastive learning (CL), which optimizes our network with three different constraints. The experimental results of three public datasets demonstrate that our method outperforms the existing state-of-the-art methods.

## 1. Introduction

Hyperspectral sensors are instruments capable of capturing the spectral information of objects as they reflect, emit, or transmit light across a continuous spectral range. In comparison to conventional multispectral or panchromatic sensors, hyperspectral sensors offer tens to hundreds of contiguous, narrow spectral bands, thereby enabling the capture of more intricate spectral features. Consequently, Hyperspectral Images (HSIs) derived from data collected by these sensors encompass a wealth of spectral information about the Earth’s surface [1]. Currently, HSIs find broad applications in fields such as forestry, geology, military reconnaissance, agriculture, and environmental monitoring [2,3]. Among the core issues in HSI research, HSI classification has long been a subject of extensive interest, requiring the prediction of categories for each pixel in HSI [4]. However, in situations where the number of labeled samples is insufficient, the data are high-dimensional, and the spectral signatures are subject to interference due to complex scenes, existing networks fail to adequately explore the intrinsic features of HSI, thereby hindering the accurate classification of pixel categories. Therefore, developing more effective HSI classification methods that can handle limited labeled samples, high dimensionality, and complex spectral signatures remains a crucial and ongoing research challenge.

Conventional methods for HSI classification that rely on spectral features mainly encompass Support Vector Machines (SVMs) [5], Random Forests (RF) [6], Rotation Forests [7], and Multinomial Logistic Regression (MLR) [8]. In pursuit of augmenting the precision of HSI classification, scholars have explored the fusion of spatial attributes with spectral data [9,10,11], culminating in the development of methodologies grounded in combined spectral-spatial characteristics. Such methods encompass the Gray Level Co-occurrence Matrix (GLCM) [12], Wavelet Transforms [13], and Gabor Filtering [14], among others, which can extract richer features from both spatial and spectral dimensions, thereby improving feature recognition rates. Furthermore, an approach known as Extended Morphological Profiles (EMP) [15,16] has been introduced, which leverages spatial contextual information through a series of morphological operations to enhance the classification of HSI. However, the aforementioned methods require the manual selection and design of critical spectral-spatial features for training the model, which necessitates that researchers have knowledge of which spectral and spatial data are most pertinent to the classification task. Consequently, these models are heavily reliant on prior knowledge and experience-based hyperparameters [17,18].

Over the recent period, deep learning paradigms, prominently featuring Convolutional Neural Networks (CNNs), have surfaced as innovative strategies for tackling HSI classification challenges. Contrasted with traditional classification techniques, CNNs excel in automatically discerning profound information from HSI, removing the need to manually design complex hyperparameters. For example, Wang et al. [19] designed an end-to-end Fast Dense Spectral-Spatial Convolution Network (FDSSC) that utilizes convolutional kernels of various sizes to effectively capture spectral-spatial information from HSI data, attaining high accuracy in classification by leveraging both spatial context and spectral information. Zhong et al. [20] proposed a supervised Spectral-Spatial Residual Network (SSRN) to capture the joint spectral-spatial information of HSI for classification, which alleviates the issue of gradient vanishing or explosion that arises as the network layers count grows. These CNN-based methods mainly adhere to the patch-based local learning framework [21,22,23]. However, dividing HSI into fixed-size overlapping patches not only introduces redundant computations but also limits the perception range of the entire network, hindering the connections between pixels beyond the patch scope. This limitation prevents the model from extracting the global information of HSI. Furthermore, deep learning approaches typically require a substantial amount of annotated data, which are often challenging to acquire in real-world scenarios because of the labor-intensive and expensive process of data annotation. This makes it difficult to obtain enough annotated examples to satisfy the requirements for classifying HSI. Therefore, this necessitates extracting sufficient information from a restricted quantity of annotated examples to optimize our network.

To address the aforementioned problems, this paper proposes an Advanced Global Prototypical Segmentation (AGPS) framework, which consists of five components: an encoder, an Atrous Spatial Pyramid Pooling-Position Attention (ASPP-PA) module, a decoder, a Fusion of Lateral Connection (FLC) structure, and an advanced global prototypical representation learning strategy. Notably, the encoder, ASPP-PA module, decoder, and FLC structure together form the feature extractor segmentation network (SegNet) of AGPS. Specifically, to address the drawbacks of patch-based models, which include the inability to capture global information and excessive computational redundancy, we propose SegNet, a segmentation network with an encoder-decoder structure based on a Fully Convolutional Network (FCN). Since its input is the entire HSI without the need to divide the HSI into fixed-size overlapping patches centered on individual pixels, and it performs one-shot forward computation and can obtain the feature map of the entire HSI, it is not limited by the patch size in terms of the network receptive field and redundant computations. To enrich the global information extracted by SegNet, we introduce an FLC structure and a multi-scale position attention module, called the ASPP-PA module. The FLC structure is responsible for fusing the low-level detail features output by the encoder with the high-level features output by the decoder, ensuring that each output feature vector of SegNet contains sufficient spectral-spatial information for accurate category prediction. The ASPP-PA module is formed by connecting the Atrous Spatial Pyramid Pooling (ASPP) module and the Position Attention (PA) module in series, where the ASPP module extracts and fuses multi-scale features, and the PA module captures the spatial dependencies of the fused feature maps. To tackle the issue of limited labeled HSI samples, we adopt a transfer learning approach to train the network and combine a global prototypical representation learning strategy [24] with supervised Contrastive Learning (CL), proposing an advanced global prototypical representation learning strategy. The learning strategy employs three different constraints to optimize the network, and since CL uses positive and negative sample pairs for training, it can extract more information from fewer labeled samples to optimize the network. The primary contributions of this article include the following:

- To capture global information, a patch-free feature extractor based on FCN is proposed. The input to the extractor is the entire HSI, and the features of the entire HSI can be obtained through one-shot forward computation. This process is similar to semantic segmentation, so we refer to the proposed feature extractor as the segmentation network (SegNet). Since SegNet takes the entire HSI as input, it does not restrict the receptive field of the network or create computational redundancies by dividing the HSI into fixed-size overlapping patches. As a result, SegNet offers a significantly larger receptive field than patch-based methods;
- Building upon the data characteristics of HSI and the network architecture of SegNet, we propose an FLC structure that fuses the rich detail features from the encoder with the semantic information in the decoder to enhance the feature representation capability of SegNet. Furthermore, we design a multi-scale position attention module called the ASPP-PA module by concatenating the ASPP module and PA module to fuse information across different scales and to allocate more attention to critical areas;
- To better adapt to few-shot scenarios, we integrate the global prototypical representation learning strategy with supervised CL and propose an advanced global prototypical representation learning strategy that learns a global prototypical representation feature vector for each class in HSI as the representative of that class and optimizes the network through a triplet constraint. Due to the incorporation of CL, the similarity between feature vectors of the same class increases, while the similarity between different classes decreases, thus enabling SegNet to map HSI to a more easily classifiable feature space.

## 2. Related Works

### 2.1. Extraction of Global Information from HSI

Existing methods for HSI classification are mostly based on local learning approaches using patches [25]. Patch-based methods first generate a patch for each pixel in the HSI, with that pixel as the center and surrounded by neighboring pixels. The shape of the patch is generally square, these patches are then input into the network to ultimately generate class labels [26]. However, patches generated from adjacent pixels are overlapping, and the overlapping regions participate in multiple computations, resulting in a large number of redundant calculations. Simultaneously, since the size of the input patches to the network is fixed, the receptive field of the network will not exceed the patch size, which will hinder the modeling of long-range pixel dependencies in HSI. Therefore, patch-based methods can only enhance the network’s receptive field by increasing the patch size, but the larger the patch, the more redundant computations are produced, leading to a significant decline in network efficiency.

FCNs are patchless methods, where the input is the entire HSI without needing to divide it into patches for each pixel, nicely avoiding the issues that come with patch-based local learning approaches. The general process is as follows: First, the entire HSI is input into the network and goes through several rounds of downsampling for feature extraction. The scale of the resulting feature maps decreases progressively with every downsampling step. Afterward, the feature maps undergo upsampling to restore them to the same size as the original HSI. At this point, the feature vector at each position in the feature map corresponds to the pixel at the respective position in the original HSI. Finally, the output feature map is passed through a convolutional layer (or a fully connected layer) to predict the label for each pixel. Consequently, some researchers have attempted to adapt existing FCNs for HSI classification tasks. Wang et al. [4] proposed a Fully Contextual Network (FullyContNets) to capture global features and introduced a scale attention module into the network, which can establish the dependence relationship between features on multiple scales. Zhuo et al. [25] proposed a Fast Patch-Free Global Learning (FPGA) framework that leverages an FCN-based encoder-decoder structure to process the entire image for extracting global information, thereby enabling fast inference. While existing FCNs have demonstrated promising performance in HSI classification, they typically require a great number of labeled samples, which are often impractical to obtain in real-world scenarios. In this work, to enable FCN to achieve remarkable performance with few labeled samples, we make the following two enhancements: (1) using transfer learning to pre-train SegNet and (2) combining SegNet with our proposed advanced global prototypical representation learning strategy, employing a metric learning approach better suited for small sample scenarios for the final classification.

### 2.2. HSI Classification in Few-Shot Scenarios

To address the issue of the declining classification accuracy of HSI in scenarios with limited labeled samples, researchers have introduced a method known as few-shot learning (FSL), which, compared to previous methods, achieves significant accuracy even in limited labeled sample scenarios [27,28]. FSL is an important branch of machine learning, aimed at solving problems with only a small amount of training samples, such as object recognition, face recognition, and image classification [29,30,31,32]. Currently, FSL primarily encompasses three approaches: model-based, metric-based, and optimization-based strategies. Among these methods, metric-based approaches are widely applied in the HSI classification domain due to their simplicity and effectiveness [33]. There are three main types of metric-based methods: Prototype Networks [34], Relation Networks [35], and Siamese Neural Networks [36].

Compared to Relation Networks and Siamese Neural Networks, Prototype Networks employ far fewer model parameters, requiring only the storage of prototypical vectors for each class rather than numerous parameters to depict complex network structures. This aspect significantly eases the training and adjustment process of Prototype Networks. Furthermore, Prototype Networks exhibit a robust capacity for managing new categories; when a new class is encountered, it is simply needed to incorporate the samples of that class into the prototypical vector space and recalculate the prototypical vectors accordingly. Therefore, researchers tend to favor Prototype Networks for few-shot HSI classification. Li et al. [37] presented a novel approach termed Deep Cross-Domain Few-Shot Learning (DCFSL), designed specifically for the classification of HSI, which innovatively integrates an adversarial domain adaptation mechanism within the FSL paradigm to tackle the issue of domain shift. However, in the prediction phase, the prototypical representation generated by DCFSL for each category is obtained by averaging a subset of labeled samples from that category, which may lead to a decrease in the effectiveness of the prototype. To address this issue, researchers have made numerous improvements to the prototype networks [38,39]. Zhang et al. [24] introduced the Global Prototypical Network (GPN), which utilizes a global prototypical representation learning strategy for training the network. This learning strategy obtains the global prototypical representation and episodic prototypical representations for each category by calculating the means of all labeled samples and randomly selected partially labeled samples, respectively. Subsequently, the global prototypical representation of each category is updated based on its similarity with the episodic prototypical representations of all categories. Since the global prototypical representation of each category aggregates information from other categories, the prototypes generated in this manner are more representative. Nonetheless, the aforementioned methods still inadequately leverage the information from limited labeled samples available. In this work, we introduce supervised CL into the global prototypical representation learning strategy and optimize the network through three different constraints.

## 3. Proposed Method

### 3.1. Overall Framework

**Figure 1:**
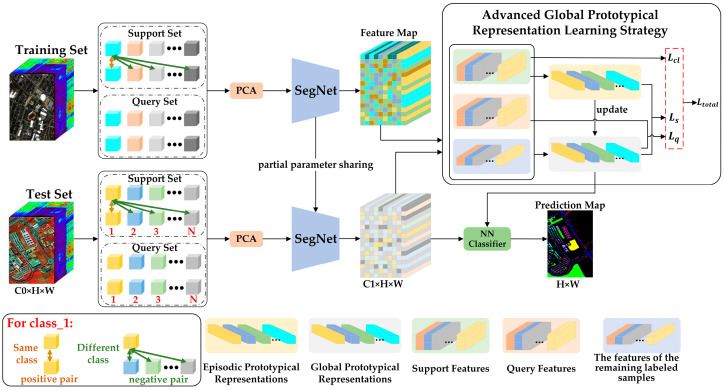
The architecture of the AGPS framework.

In Figure 1, we illustrate the implementation process of the AGPS framework. We employ transfer learning to train the feature extraction network, SegNet. Specifically, we first pre-train the network using a source domain dataset, i.e., the training set, which has sufficient labeled samples. Subsequently, we fine-tune the pre-trained network using a target domain dataset, i.e., the test set, which has only a few annotated examples.

In the AGPS framework, SegNet serves as the feature extractor, responsible for extracting the spatial-spectral features of HSI. It generates a feature vector for each pixel in the HSI. The resultant feature map retains the same dimensions as the initial HSI, and each feature vector in the feature map corresponds to the pixel at the respective position in the original HSI. The advanced global prototypical representation learning strategy optimizes SegNet through three different constraints, which are realized via three different loss functions. Specifically, in each episode, labeled samples are randomly selected from the dataset and divided into support and query sets (in Figure 1, the cubes of the same color in the support set and the query set represent samples from the same class). By taking the mean of the feature vectors of all labeled samples for each category in the dataset, we obtain the global prototypical representation feature vector for each category. Similarly, by taking the mean of the feature vectors of the samples for each category in the support set, we obtain the episodic prototypical representation feature vector for each category. The cross-entropy loss between the episodic prototypical representations and the global prototypical representations is calculated to obtain the support set loss $L_{s}$. Subsequently, the global prototypical representations are updated based on their similarity to the episodic prototypical representations. The query set loss $L_{q}$ is obtained by calculating the cross-entropy loss between the query set samples and the updated global prototypical representations. The contrast loss $L_{cl}$ is obtained by calculating the noise contrast estimation loss among the support set samples. Therefore, the total loss $L_{total}$ in an episode is the sum of $L_{s}$, $L_{q}$, and $L_{cl}$. The network parameters are finally updated using the total loss $L_{total}$.

After the network fine-tuning training is completed, we adopt the nearest neighbor (NN) algorithm for classification. Specifically, the feature vectors of the unlabeled samples after SegNet mapping are directly compared with the trained global prototypical representation of each class by computing their similarity. The category of the global prototypical representation most similar to this sample is chosen as the predicted category of this sample. In the remaining subsections, we will conduct a detailed analysis of each component of the AGPS framework.

### 3.2. SegNet

**Figure 2:**
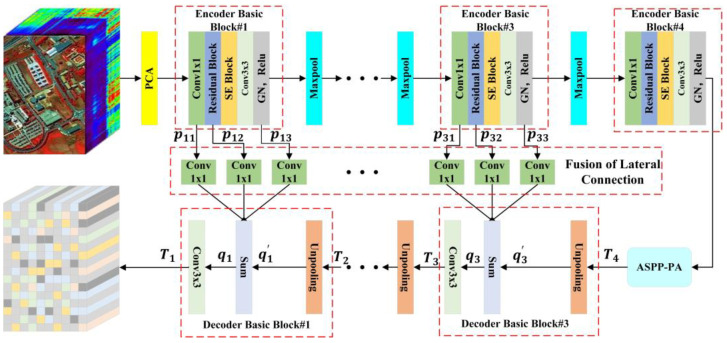
Illustrations of SegNet.

In the AGPS framework, we design a feature extractor SegNet based on the encoder-decoder architecture framework. Its objective is to acquire a transformation function that translates input HSI from its original representation to a feature space that facilitates classification. The original HSI is directly input into SegNet after dimensionality reduction using Principal Component Analysis (PCA) technology. SegNet adopts an encoder-decoder network structure. Among them, the encoder’s role is analogous to most feature extraction networks based on patch-based methods, primarily tasked with extracting spatial-spectral information from the HSI. Since its input is the entire HSI, the receptive field of our model will not be limited by the size of the patch, thus realizing the idea of replacing the fixed-size image patch with the receptive field of the model. In the encoder, we use max pooling downsampling operations to compress the feature maps. This compression process helps to increase the network’s perceptual horizon and capture a wider range of context information, but it can also result in the vanishing of some fine details. The feature map produced by the encoder is significantly smaller than the original HSI, which will result in the inability to classify each pixel of HSI based solely on the feature map output by one-shot forward computation of the encoder. Therefore, SegNet adds a decoder after the encoder, which restores the size of the feature map through the upsampling operation in the decoder. Finally, the feature vector of each pixel in HSI can be obtained through one-shot forward computation, avoiding the computational redundancy caused by multiple calculations. In addition, we also add an FLC structure and ASPP-PA module in SegNet to enrich the global information extracted. The structure of SegNet is shown in Figure 2, and each module will be described in detail below.

#### 3.2.1. Encoder

The encoder in SegNet adopts a modular design. As shown in Figure 2, the encoder network contains four encoder basic blocks and three rounds of max pooling downsampling. The encoder basic block comprises a 1 × 1 convolutional layer, a Residual Block, a channel attention module called a Squeeze-and-Excitation (SE) Block, and a 3 × 3 convolutional layer. Here, the 1 × 1 convolutional layer is used to adjust the feature map’s depth. The Residual Block is responsible for extracting spatial information from HSI, and its residual structure effectively to effectively prevent the gradient vanishing problem and enhance the feature extraction capability. The SE block, proposed by Hu et al. [40], is introduced here primarily to focus the network more on important channel information, thereby better extracting spectral information from HSI. Finally, the 3 × 3 convolutional layer is used to integrate feature information. It is important to note that because SegNet’s input is a complete HSI, batch normalization (BN) cannot be used. Instead, we opt for Group Normalization (GN) as an alternative to BN, which offers equivalent efficacy to BN and is independent of the batch size [25].

#### 3.2.2. Fusion of Lateral Connection (FLC) Structure

In the decoder, the feature maps output after upsampling possess deep-level features, but these are insufficient for classifying each pixel of HSI. However, the feature maps output by shallow convolution layers in the encoder contains a wealth of low-level detail features. Integrating these detailed features with the deep-level features can significantly enhance the overall network’s classification accuracy. Unlike ordinary 3D images, each pixel in a HSI encases a large amount of information. Therefore, to enable the feature maps output by the upsampling in the decoder to blend more bottom-layer detail information, the FLC structure merges the three feature maps output by the Encoder Basic Blocks before each downsampling, with the corresponding upsampling output feature maps. Here, the 1 × 1 convolution layer is utilized to adjust the dimensionality of the feature maps output by the Encoder Basic Block, ensuring they are consistent in shape with the corresponding upsampling output feature maps. The FLC structure can be described as:

(1) $$q_{i}=\sum_{j=1}^{3}\;conv\left(p_{ij}\right)+q_{i}^{\prime }\;i=1,2,3,$$

where $q_{i}$ denotes the fused feature map in Decoder Basic Block#i, $q_{i}^{\prime }$ represents the output of the upsampling layer in Decoder Basic Block#i, conv represents the convolution operation and $p_{ij}$ denotes the output of the jth module in Encoder Basic Block#i.

#### 3.2.3. Atrous Spatial Pyramid Pooling-Position Attention (ASPP-PA) Module

The paper proposes a novel multi-scale position attention module called ASPP-PA, which combines the ASPP module with the PA module. The ASPP module employs dilated convolutions with varied dilation factors to grasp context across multiple spatial scales. Compared to regular convolutions, dilated convolutions introduce zero values in the convolution kernels, which increases the network’s perception scope without augmenting either the parameter count or computational cost. The PA module employs self-attention mechanisms to discern the spatial relationships between features from diverse spatial positions.

(1) Atrous Spatial Pyramid Pooling (ASPP) Module

**Figure 3:**
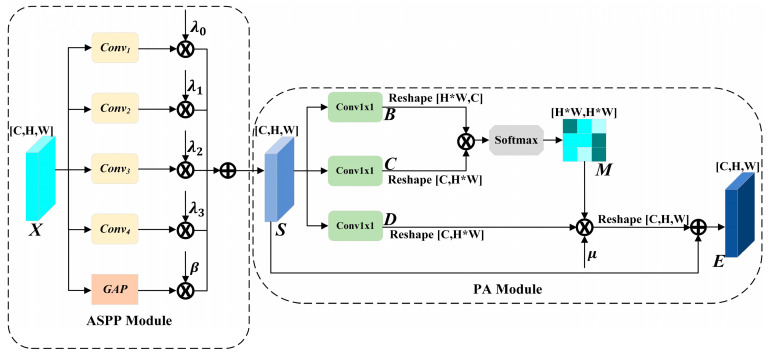
ASPP-PA Module.

The structure of ASPP is shown in Figure 3, where ASPP utilizes dilated convolution operations with different dilation factors and global average pooling (GAP) to capture spatial information at multiple scales. These features are then consolidated via summation. Given a feature map $X\in R^{C\times H\times W}$, the feature map S with multi-scale information after ASPP processing is represented as follows:

(2) $$S=\sum_{i=0}^{3}\;\lambda_{i}{\cdot Conv}_{i}(X)+\beta \cdot GAP(X),$$

where $S\in R^{C\times H\times W}$, ${Conv}_{i}\;(i=0,1,2,3)$ represents the four dilated convolution operations, GAP denotes the global average pooling operation, and $\lambda_{i}\;(i=0,1,2,3)$ and β are five learnable parameters, all initialized to 1. Due to the learnable parameter settings, the ASPP module can assign greater weights to important scale information when extracting multi-scale information.

In the experiment, the number of dilated convolutions is set to 4, which achieves a good balance between computational efficiency and accuracy [41]. It should be noted that, unlike ordinary convolution, dilated convolution can increase the receptive field size obtained from the original image without reducing spatial resolution. However, when performing dilated convolution calculations, not all pixels in the feature map participate in the calculations due to the insertion of 0-pixel values. To maximize the retention of information completeness at various scales, we set the dilation factors to [1,2,3,5].

(2) Position Attention (PA) Module

To further integrate S, we sequentially attach a PA module after the ASPP module to obtain the spatial dependencies among features at different locations within S. The feature map S, which integrates multi-scale information, is initially processed by a convolutional layer and subsequently undergoes a shape transformation to yield two novel feature mappings, B and C, where $B,C\in R^{C\times N}$. Here, N = H∗W represents the total pixels count of S. Subsequently, the new feature mapping matrices B and C are multiplied, and the resulting product is then normalized to produce the spatial position attention map $M\in R^{N\times N}$:

(3) $$M_{ji}=\frac{exp(B_{i}\cdot C_{j})}{\sum_{i=1}^{N}\;exp(B_{i}\cdot C_{j})},$$

where $M_{ji}$ represents the dependency relationship between the ith feature and the jth feature in S, and $B_{i}$ and $C_{j}\in R^{C\times 1}$ represent the feature vector at the ith position in B and the feature vector at the jth position in C, respectively. The more similar the feature representations of these two positions are, the larger the value of $M_{ji}$ between the ith feature and the jth feature in S.

Subsequently, we input S into the convolutional layer to generate a new feature map $D\in R^{C\times N}$, where it should be noted that although B, C and D are generated in the same way, they are not entirely identical since the 1 × 1 convolutional parameters used to generate B, C and D are different. By multiplying the new feature map D with the spatial position attention map M and the variable parameter μ, and subsequently adding this product to S, we can attain the final output $E\in R^{C\times H\times W}$:

(4) $$\begin{array}{c} E_{j}=\mu \sum_{i=1}^{N}\;(M_{ji}{\cdot D}_{i})+S_{j} \end{array},$$

where μ is a learnable parameter, with its initial value set to 1. $E_{j}$, $D_{i}$ and $S_{j}\in R^{C\times 1}$ represents the feature vector at the jth position of the final feature map E, the feature vector at the jth position of the feature map D and the feature vector at the jth position of the feature map S, respectively. According to Equation (4), it is the weighted sum of the feature vectors at all positions in S and the feature vector at the jth position. Therefore, the final feature map E has a global contextual view.

#### 3.2.4. Decoder

The decoder in this work differs from the decoders used in previous FCNs, as it does not directly predict pixel categories but rather serves as part of the feature extractor. Similar to the encoder network, the decoder network also adopts a modular design, consisting of three basic decoder blocks, as shown in Figure 2. The process of each decoder module is as follows: the input feature map is first upsampled by a factor of 2 using bilinear interpolation, then it is fused with the output of the FLC that has more detailed spectral-spatial information. Finally, a 3 × 3 convolutional layer is used to integrate the fused feature information. The decoder can be described as:

(5) $$T_{i}={conv}_{3\times 3}\left(\sum_{j=1}^{3}\;conv\left(p_{ij}\right)+Unpooling(T_{i+1})\right)\;i=1,2,3,$$

where $T_{i}$ represents the output of Decoder Basic Block#i, $T_{4}$ represents the feature map processed by the ASPP-PA module, $p_{ij}$ represents the output of the jth module in Encoder Basic Block#i, Unpooling represents the 2× upsampling operation, conv represents for a 1 × 1 convolutional layer, and ${conv}_{3\times 3}$ represents a 3 × 3 convolutional layer.

### 3.3. Advanced Global Prototypical Representation Learning Strategy

The AGPS framework adopts an episodic training method, where the network is trained using only the selected support set and query set within each episode. The training method for the training set and the test set is exactly the same, requiring the partitioning of labeled data into support and query sets within each episode before using them to train the network. It is important to note that since we utilize a transfer learning approach to pre-train the network, there is a sequential order in training the training set and the test set. Initially, the network is pre-trained using the training set, which has a sufficient number of labeled samples. After the network pre-training is completed, the parameters of the Encoder Basic Block #1 and #2 in the encoder of SegNet are retained. Then, the network is fine-tuned using the test set data. The steps for fine-tuning the network are completely identical to those of pre-training, except that the parameters of the basic blocks #1 and #2 in the encoder of SegNet are no longer updated.

The objective of the advanced global prototypical representation learning strategy is to optimize the feature extraction network and generate a global prototypical representation feature vector for each category that can represent this category. This strategy is completely the same in both the training and test sets. In Section 3.3.1 and Section 3.3.2 below, we will illustrate the implementation details of the global prototypical representation learning strategy and how CL is incorporated, using an example of episodic training from the test set.

#### 3.3.1. Global Prototypical Representation Learning Strategy

We first divide the labeled samples in the test set into a query set and a support set. Let $C_{all}=\{c_{1},c_{2},c_{3},\ldots ,c_{N}\}$ be the label set of all classes. We randomly select K samples (K is set to 2 in the experiments) from each class to form the support set $S=\{(x_{i},y_{i}){\}}_{i=1}^{N\times K}$. Then, we randomly select M samples (M is set to 2 in the experiments) from the remaining samples of each class to serve as the query set $Q={\left\{\left(x_{j},y_{j}\right)\right\}}_{j=1}^{N\times M}$. The samples in the support set and the query set are disjoint.

In an episode, the global prototypical representation $g\left(c_{i}\right)$ for the class $c_{i}$ can be expressed as:

(6) $$g(c_{i})=\frac{\sum_{l=1}^{Num}\;f_{\theta }(p_{il})}{\mathrm{Num}},$$

where $g\left(c_{i}\right)\in R^{d\times 1}$ is the sample feature mean of class $c_{i}$ in the test set. The $p_{il}$ represents the labeled sample in class $c_{i}$, where l represents the sample serial number. Num is the total count of labeled samples in each category, and $f_{\theta }$ is the mapping function of the feature extractor. The global prototypical representations of all categories are combined into a matrix $G=\{g\left(c_{1}\right),g\left(c_{2}\right),g\left(c_{3}\right),\ldots ,g\left(c_{N}\right)\}$, $G\in R^{d\times N}$.

In the same episode, the episodic prototypical representation $e(c_{i})$ of class $c_{i}$ can be expressed as:

(7) $$e(c_{i})=\frac{\sum_{l=1}^{K}\;f_{\theta }(s_{il})}{K},$$

where $s_{il}$ represents the labeled sample in class $c_{i}$ of the support set. K is the number of labeled samples in each category in the support set.

Subsequently, we compute the similarity between $e(c_{i})$ of and the global prototypical representations of all classes:

(8) $$h_{i}^{j}=\frac{e(c_{i})\cdot g(c_{j})}{\left||e(c_{i})||\cdot ||g(c_{j})|\right|}\;j=1,2,\ldots ,N,$$

where $h_{i}^{j}$ represents the similarity between $e(c_{i})$ and $g(c_{j})$, $\left||e(c_{i})|\right|$ and $\left||g(c_{j})|\right|$ are the moduli of $e(c_{i})$ and $g(c_{j})$, respectively. We combine $h_{i}^{j}$, j=1,2,…,N into a matrix $H_{i}={[h}_{i}^{1},h_{i}^{2},h_{i}^{3},\cdots ,h_{i}^{N}{]}^{T}$, and $H_{i}\in R^{N\times 1}$. $H_{i}$ is the similarity matrix between $e(c_{i})$ and the global prototypical representations of all classes. We then transform $H_{i}$ into a probability distribution $P_{hi}={[p}_{hi}^{1},p_{hi}^{2},p_{hi}^{3},\cdots ,p_{hi}^{N}{]}^{T}$, and $P_{hi}\in R^{N\times 1}$, using the softmax function. Thus, the cross-entropy loss for class $c_{i}$ in the support set can be expressed as:

(9) $$L_{s}^{i}=-\sum_{j=1}^{N}\;y_{j}log(p_{hi}^{j}),$$

where $p_{hi}^{j}$ denotes the normalized value of $h_{i}^{j}$. And $y_{j}$ is the true probability, $y_{j}=1$ only when j=i, and $y_{j}=0$ otherwise. The total loss $L_{s}$ for the support set can be represented as:

(10) $$L_{s}=\frac{1}{N}\sum_{i=1}^{N}\;L_{s}^{i},$$

Subsequently, we update $g(c_{j})$ using the probability distribution $P_{hi}$. The formula for $g_{\mathrm{update}}(c_{i})$ is as follows:

(11) $$g_{\mathrm{update}}(c_{i})={G\times P}_{hi},$$

It is obtained by calculating the attention weights through attention mechanism based on $e(c_{i})$ and G, multiplying the attention weights with the corresponding global prototypical representations, and then aggregating the results. Therefore, the $g_{\mathrm{update}}(c_{i})$ contains information on all classes. Similarly, the updated global prototypical representations for all classes can be obtained as $G_{\mathrm{update}}=\{g_{\mathrm{update}}\left(c_{1}\right),\;g_{\mathrm{update}}\left(c_{2}\right)$, $g_{\mathrm{update}}\left(c_{3}\right)$, $...,\;g_{\mathrm{update}}\left(c_{N}\right)\}$, $G_{\mathrm{update}}\in R^{d\times N}$.

The similarity between the sample $q_{il}$ from class $c_{i}$ in the query set and the updated global prototypical representation of each class can be expressed as:

(12) $$w_{il}^{j}=\frac{f_{\theta }\left(q_{il}\right)\cdot g_{\mathrm{update}}\left(c_{j}\right)}{\left||f_{\theta }\left(q_{il}\right)||\cdot ||g_{\mathrm{update}}\left(c_{j}\right)|\right|}\;j=1,2,\ldots ,N,$$

where $w_{il}^{j}$ signifies the similarity between the feature vector obtained after mapping $q_{il}$ and $g_{\mathrm{update}}\left(c_{j}\right)$. We combine $w_{il}^{j}$, j=1,2,…,N into a matrix $W_{il}={[w}_{il}^{1},w_{il}^{2},w_{il}^{3},\cdots ,w_{il}^{N}{]}^{T}$, $W_{il}\in R^{N\times 1}$. $W_{il}$ is the similarity matrix between $q_{il}$ and the updated global prototypical representations of all classes. $W_{il}$ is then transformed into a probability distribution $P_{wil}={[p}_{wil}^{1},p_{wil}^{2},p_{wil}^{3},\cdots ,p_{wil}^{N}{]}^{T}$, $P_{wil}\in R^{N\times 1}$, using the softmax function. The cross-entropy loss for sample $q_{il}$ from class $c_{i}$ in the query set can be expressed as:

(13) $$L_{q}^{il}=-\sum_{j=1}^{N}\;y_{j}log(p_{wil}^{j}),$$

where $p_{wil}^{j}$ denotes the normalized value of $w_{il}^{j}$, $y_{j}$ is the true probability, $y_{j}=1$ only when j=i, and $y_{j}=0$ otherwise. The total loss for the query set $L_{q}$ can be expressed as:

(14) $$L_{q}=\frac{1}{M\cdot N}\sum_{i=1}^{N}\;\sum_{l=1}^{M}\;L_{q}^{il},$$

where M⋅N represents the total number of samples in the query set.

#### 3.3.2. Introduce Supervised CL

To obtain more information from a few labeled samples and optimize the feature extraction network, this paper introduces supervised CL into the global prototypical representation learning strategy. By computing the contrastive loss on the support set samples, ensuring that the feature vectors of samples mapped through the SegNet have increased similarity within the same class and decreased similarity between different classes. This maps the HSI to a feature space that facilitates easier classification.

In the AGPS framework, the supervised CL is applied to the support set samples. We divide the support set samples into two groups, denoted as $A=\left\{x_{1}^{1},x_{1}^{2},x_{1}^{3},\ldots ,x_{1}^{N}\right\}$, and $B=\{x_{2}^{1},x_{2}^{2},x_{2}^{3},\ldots ,x_{2}^{N}\}$, where the subscript a in $x_{a}^{b}$ signifies group membership where a=1 denotes Group A and a=2 denotes Group B, the superscript b in $x_{a}^{b}$ represents the class index. Each class has only one sample in each group, and the two groups of samples are mutually exclusive. In the supervised CL, two samples sharing the same class can be assembled into a positive pair, while a negative pair is constituted by two samples belonging to different categories. Clearly, for the sample $x_{1}^{1}$ in group A, the only sample in group B that can form a positive pair with it is $x_{2}^{1}$.

In this paper, CL is implemented using the InfoNCE (Noise Contrastive Estimation) loss function. The loss function $L_{x_{1}^{1},x_{2}^{1}}$ for a positive sample pair $x_{1}^{1}$ and $x_{2}^{1}$, can be expressed as follows:

(15) $$L_{x_{1}^{1},x_{2}^{1}}=-log\frac{exp({s(f}_{\theta }\left(x_{1}^{1}\right),f_{\theta }\left(x_{2}^{1}\right))/\tau )}{\sum_{i=1}^{N}\;exp({s(f}_{\theta }\left(x_{1}^{1}\right),f_{\theta }\left(x_{2}^{i}\right))/\tau )},$$

where ${s(f}_{\theta }\left(x_{1}^{1}\right),f_{\theta }\left(x_{2}^{1}\right)$ is used to calculate the similarity between $f_{\theta }\left(x_{1}^{1}\right)$ and $f_{\theta }\left(x_{2}^{1}\right)$, and τ is the temperature coefficient, which is set to 0.5 in the experiments [33]. Due to the existence of the denominator in the loss function, $L_{x_{1}^{1},x_{2}^{1}}\neq L_{x_{2}^{1},x_{1}^{1}}$. Therefore, the total contrastive $L_{cl}$ in an episode can be represented as:

(16) $$L_{cl}=\frac{1}{2N}\sum_{i=1}^{N}\;(L_{x_{1}^{i},x_{2}^{i}}+L_{x_{2}^{i},x_{1}^{i}}),$$

The total loss $L_{total}$ in an episode can then be represented as:

(17) $$L_{total}={\alpha L}_{cl}+(1-\alpha )\cdot (L_{q}+L_{s})/2,$$

where α is a hyperparameter, and a larger value of α will give more weight to the contrastive loss $L_{cl}$ during backpropagation. We choose the Adam optimizer to optimize the network, with an initial learning rate set to 0.0001.

## 4. Experimental Results and Analysis

In this experiment, we did not directly use the test dataset for training the network. Instead, we employed a transfer learning approach. We first performed pre-training on the network using the training dataset and then fine-tuned it with labeled examples from the test dataset. The test dataset has only a few labeled samples. Furthermore, the classes in the training dataset do not necessarily have to be the same as those in the test dataset.

### 4.1. Experimental Datasets

**Figure 4:**
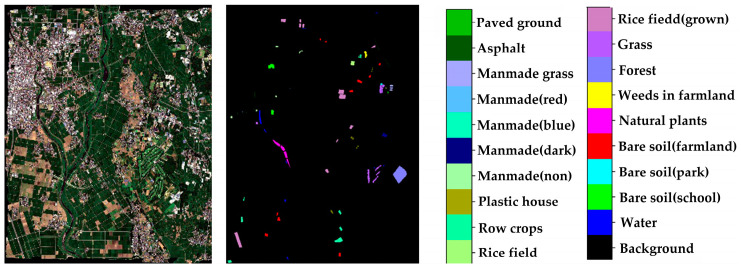
Chikusei data set.

**Figure 5:**
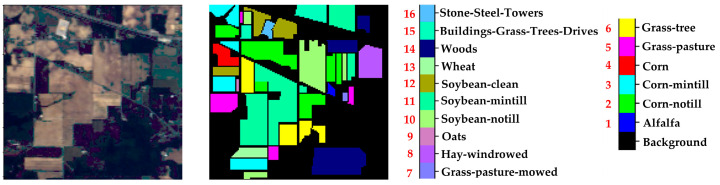
IP data set. The black font indicates the category name, and the red font indicates the category serial number.

**Figure 6:**
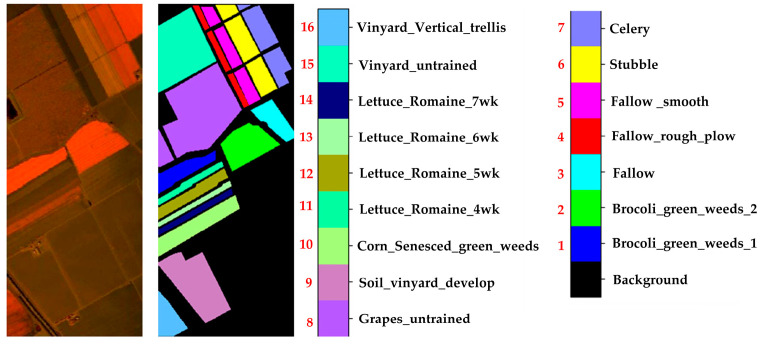
SA data set. The black font indicates the category name, and the red font indicates the category serial number.

**Figure 7:**
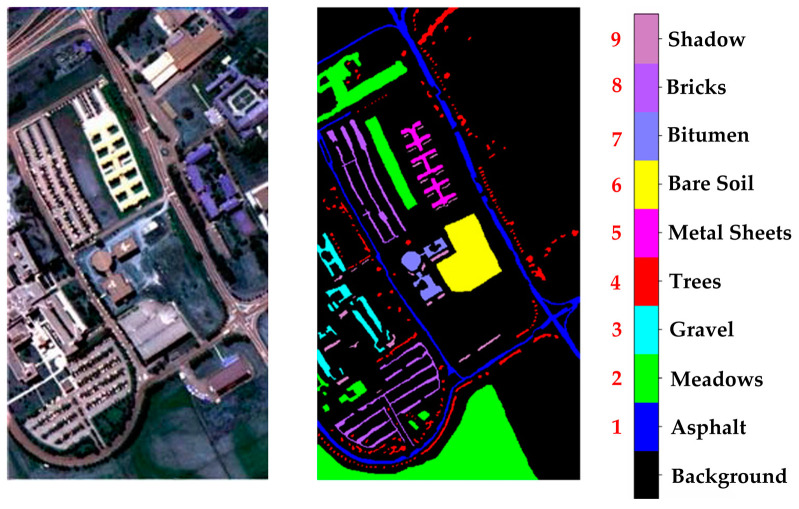
UP data set. The black font indicates the category name, and the red font indicates the category serial number.

1. Training Dataset:
  - Chikusei Dataset: The spectral range of this dataset is 343–1080 nm, with a spatial resolution of approximately 2.5 m. This dataset size is 2571 × 2335, consisting of 128 spectral bands. The Chikusei dataset is divided into 19 land cover classes. Figure 4 shows the false-color image and the ground truth of the Chikusei dataset.
2. Test Dataset:
  - Indian Pines (IP) Dataset: The spectral range of this dataset is 400–2500 nm, with a spatial resolution of approximately 20 m. This dataset size is 145 × 145, consisting of 200 spectral bands. This dataset contains 16 land cover classes. Figure 5 shows the false-color image and the ground truth of the Indian Pines dataset.
  - Salinas (SA) Dataset: The spectral range of this dataset is 400–2500 nm, with a spatial resolution of approximately 3.7 m. This dataset size is 512 × 217, consisting of 224 spectral bands. After removing the bands with severe water vapor absorption, there are 204 bands left. This dataset contains 16 land cover classes. Figure 6 shows the false-color image and the ground truth of the Salinas dataset.
  - University of Pavia (UP) Dataset: The spectral range of this dataset is 430–860 nm, with a spatial resolution of approximately 1.3 m. This dataset size is 610 × 340, consisting of 115 original bands. After removing 12 noisy bands, there are 103 bands left. This dataset contains 9 land cover classes. Figure 7 shows the false-color image and the ground truth of the University of Pavia dataset.

### 4.2. Experimental Settings

1. Running Platform: The experiments were conducted on a computer with an Intel^®^ Core^TM^ i7-6700K CPU @ 4.00 GHz, 32 GB of RAM, and an NVIDIA TITAN X (Pascal) 12 GB graphics card (Santa Clara, CA, USA). The neural network framework Pytorch 1.9.1 was used for training and testing.
2. Evaluation Metrics: The overall accuracy (OA), Kappa coefficient, and average accuracy (AA) were used for evaluation. Here, OA represents the ratio of correctly classified pixels to the total number of test pixels in HSI. AA represents the average accuracy of all classes. The Kappa coefficient is used to measure the consistency between the classification result of the hyperspectral data set and the actual effect, with a range of 0 to 1. A value of 1 indicates complete consistency, a value greater than 0.75 indicates satisfactory consistency, and a value less than 0.4 indicates less than ideal consistency [42]. The larger the values of these three evaluation metrics, the better the model performance. All experiments were conducted ten times, and the average values were taken.

### 4.3. Analysis of Hyperparameters

**Figure 8:**
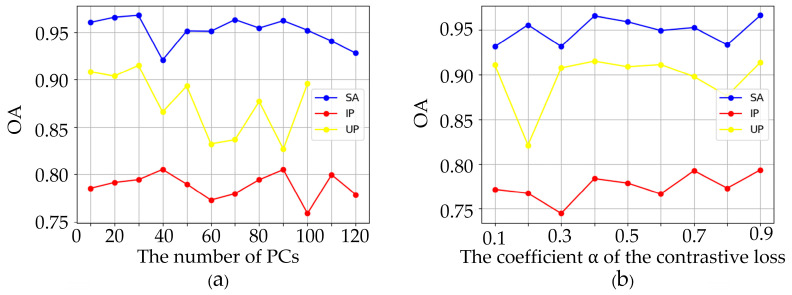
Evolution of OA as a function of (**a**) PCs, (**b**) α, where the blue curve represents the SA dataset, the red curve represents the IP dataset, and the yellow curve represents the UP dataset.

We first analyzed the impact of the number of principal components (PCs) preserved after PCA dimensionality reduction on the OA. The number of dimensions of the pre-training dataset Chikusei is 128, so even if the number of dimensions of the SA and IP datasets is greater than 120, we still set the maximum number of PCs of the SA and IP datasets to 120. The number of dimensions of the UP dataset is 103, so we set the maximum number of PCs of the UP dataset to 100. The variation of OA with the number of PCs is shown in Figure 8a. When the number of PCs is 30, the OA of the UP and SA datasets can reach the maximum value. Although the OA of the IP dataset can reach the maximum value when the number of PCs is 40, to ensure that the number of PCs can achieve better results on all three datasets, we set the number of PCs to 30.

Next, we analyzed the impact of the coefficient α of the contrast loss $L_{cl}$ on the OA. We set the coefficient α of the three datasets to {0.1, 0.2, 0.3, …, 0.9}, and the OA varies with the coefficient α as shown in Figure 8b. We can see that when the value of the coefficient α is set to 0.9, the OA in all three datasets is higher than the other values of α. Therefore, in the subsequent experiments, we set the value of the coefficient α to 0.9.

### 4.4. Analysis of Few-Shot Classification Performance in the AGPS Framework

To verify the effectiveness of our method under few-shot scenario, we considered seven methods, including SVM [5], 3-D CNN [43], FPGA [25], DFSL [27], DFSL + SVM [27], DFSL + NN [27], DCFSL [37], CMTL [44], S3Net [45], CRSSNet [42], with their parameters set to the recommended values. SVM is a traditional method, 3-D CNN and FPGA are classical deep learning methods, DFSL, DFSL + SVM, and DFSL + NN are early deep learning FSL methods that combine traditional methods, and DCFSL, CMTL, CRSSNet, and S3Net are the state-of-the-art FSL methods. Among them, only the FPGA and our method are patch-free, whereas the remaining methods are patch-based. For each category, we randomly select five samples to train the network, and the remaining samples are used as unlabeled samples to evaluate the classification accuracy of the models.

**Figure 9:**
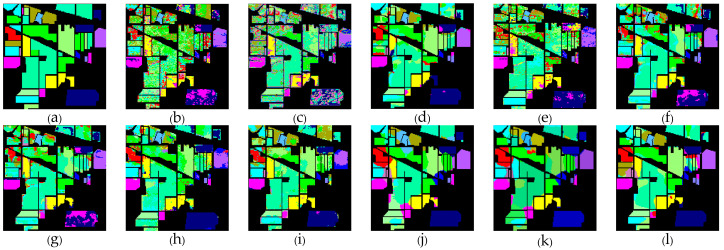
IP (**a**) Ground-truth. (**b**–**l**) Classification maps for different classifiers. (**b**) SVM. (**c**) 3D-CNN. (**d**) FPGA. (**e**) DFSL. (**f**) DFSL + SVM. (**g**) DFSL + NN. (**h**) DCFSL. (**i**) CMTL. (**j**) S3Net. (**k**) CRSSNet. (**l**) AGPS.

**Figure 10:**
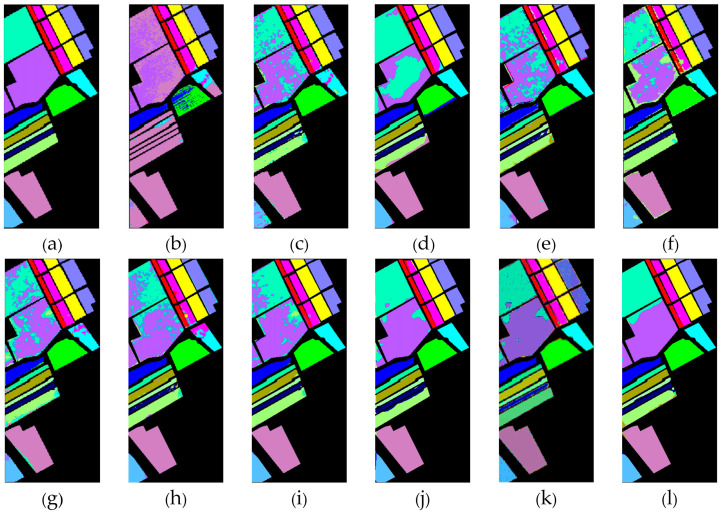
SA (**a**) Ground-truth. (**b**–**l**) Classification maps for different classifiers. (**b**) SVM. (**c**) 3D-CNN. (**d**) FPGA. (**e**) DFSL. (**f**) DFSL + SVM. (**g**) DFSL + NN. (**h**) DCFSL. (**i**) CMTL. (**j**) S3Net. (**k**) CRSSNet. (**l**) AGPS.

**Figure 11:**
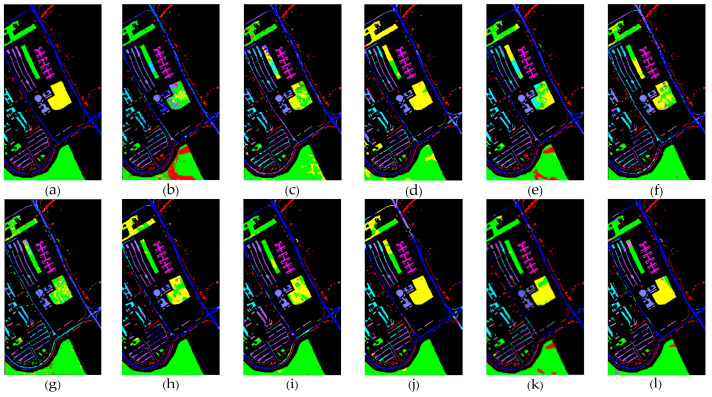
UP (**a**) Ground-truth. (**b**–**l**) Classification maps for different classifiers. (**b**) SVM. (**c**) 3D-CNN. (**d**) FPGA. (**e**) DFSL. (**f**) DFSL + SVM. (**g**) DFSL + NN. (**h**) DCFSL. (**i**) CMTL. (**j**) S3Net. (**k**) CRSSNet. (**l**) AGPS.

Table 1, Table 2 and Table 3 show the classification results of the aforementioned methods for each class on the IP, SA, and UP datasets, respectively. It can be observed that our proposed AGPS framework achieves the best OA and Kappa across all three datasets. Compared to traditional methods (SVM), deep learning methods (3D-CNN, FPGA), and early FSL methods (DFSL + NN, DFSL + SVM, DFSL), the AGPS framework demonstrates a significant improvement in all three datasets, with OA increases of 10.11–33.13% on the IP dataset, 9.56–15.9% on the SA dataset, and 11.45–26.96% on the UP dataset. Even compared to the latest FSL methods (S3Net and CRSSNet), our proposed method shows a slight improvement on the IP and SA datasets, and the OA improvement of around 3–4% on the UP dataset. Figure 9, Figure 10 and Figure 11 provide a more intuitive visualization of the classification maps generated by different methods, where the prediction map produced by the AGPS framework is most similar to the Ground-truth map. This demonstrates the superiority and stability of the AGPS framework in terms of classification performance in few-shot scenarios.

From the perspective of the entire dataset, it can be observed that the accuracy of different models on the SA dataset and UP dataset is significantly higher than that on the IP dataset. We believe that the primary reason for this disparity is the difference in spatial resolution among the datasets. For the SA and IP datasets, the spatial resolutions are 3.7 m and 1.3 m per pixel, respectively, while the IP dataset has a spatial resolution of 20 m per pixel. Higher spatial resolution implies that each pixel covers a smaller area of the ground surface, allowing for the capture of more detailed features of the terrain, which is crucial for predicting the category of each pixel. Regarding the SA dataset and the UP dataset, although their spatial resolutions are similar, the UP dataset contains only 103 spectral bands, ranging from 430 to 860 nm. In contrast, the SA dataset has 204 spectral bands, ranging from 400 to 2500 nm. This means that each pixel in the SA dataset contains more spectral information compared to the UP dataset, thereby resulting in higher classification accuracy.

For the specific classes, the accuracy of the “Soybean Notill (No. 10)”, “Soybean Mintill (No. 11)”, “Soybean Clean (No. 12)” in the IP dataset, and the “Bare soil (No. 6)” in the UP dataset is significantly higher than that of patch-based methods. We believe this is because, compared to other classes, the classification of these four classes relies more heavily on information from distant pixels, while patch-based methods are limited by the input patch size, restricting the network’s ability to capture long-range pixel relationships. On the other hand, for the “Corn Notill (No. 2)”, “Corn Mintill (No. 3)”, “Grass Pasture (No. 5)” in the IP dataset, and the “Tress (No. 4)” in the UP dataset, our method has lower accuracy compared to S3Net and CRSSNet. We attribute this to the factor that the classification of these four classes relies more on information from neighboring pixels, and the feature extraction networks of S3Net and CRSSNet adopt a Siamese network architecture with a differential input strategy, which gives them a stronger ability to extract local features compared to the AGPS framework that uses a SegNet encoder-decoder architecture as the feature extractor.

### 4.5. Analysis of Inference Speed and Computational Cost in the AGPS Framework

To validate the superior inference speed and computational cost of the AGPS framework, we selected three state-of-the-art methods: CRSSNet, S3Net, and DCFSL for comparison with the AGPS framework. We randomly selected five labeled samples per class from three datasets for training the models. After training, we used the trained models to predict the categories of the remaining unlabeled samples and compared the inference speed of different methods by measuring the time it takes for the models to make predictions. Additionally, we assessed the theoretical computational cost using gigaflops per second (GFLOPs) as the metric (noting that, due to different methods employing different prediction strategies, for consistency in calculating costs, we only calculated the computational cost for mapping unlabeled samples to the feature space).

Table 4 presents the inference time and computational costs of the four methods across three datasets. It can be seen that our method incurs significantly lower computational overhead compared to CRSSNet, S3Net, and DCFSL. This is because the patch-based methods used in CRSSNet, S3Net, and DCFSL result in a large amount of computational redundancy due to the overlapping of image patches. In contrast, our method inputs the entire HSI into the network at once and performs one-shot forward computation, thereby avoiding computational redundancy. It is worth noting that in our method, although the computational cost for the UP dataset is higher than that for the SA dataset, the actual inference time for UP is faster than SA. This discrepancy arises because, despite the SA dataset having smaller dimensions than the UP dataset, it requires predictions for a larger number of pixel points, which consequently leads to an increase in inference time.

### 4.6. Analysis of Feature Separability

**Figure 12:**
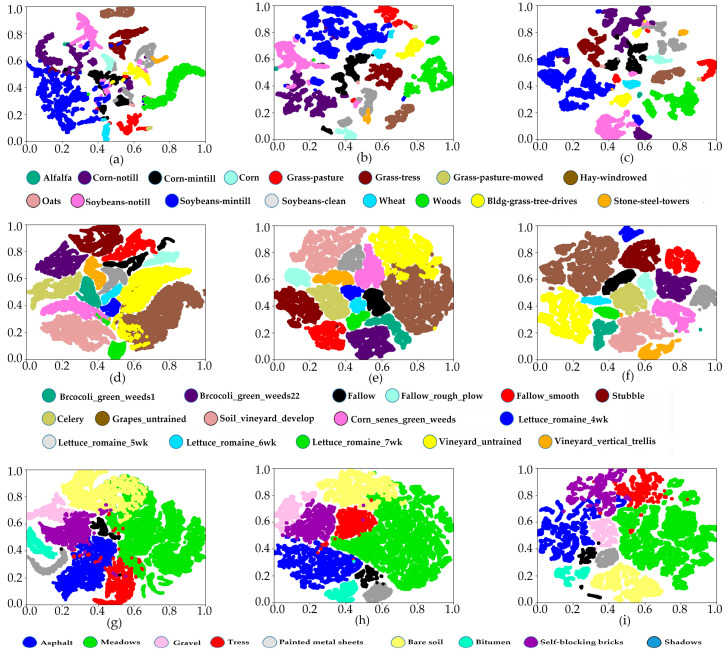
Feature separability for different methods in the three datasets. (**a**) S3Net, (**b**) CRSSNet, and (**c**) AGPS for IP. (**d**) S3Net, (**e**) CRSSNet, and (**f**) AGPS for SA. (**g**) S3Net, (**h**) CRSSNet, and (**i**) AGPS for UP.

To visually reflect the separability of different feature maps, as shown in Figure 12, we used the t-SNE method to transform the feature maps output by S3Net, CRSSNet, and our proposed method on the three datasets into a two-dimensional space.

On the IP dataset, for the categories of “Soybean-notill”, “Soybean-mintill”, and “Soybean-clean”, the feature overlap area of our method is significantly less than that of CRSSNet and S3Net. Correspondingly, our method achieves much higher classification accuracy for these three categories compared to CRSSNet and S3Net. On the SA dataset, CRSSNet and S3Net methods confuse the features belonging to the “Gaspes-Untrained” and “Vinyard-Untrained” categories, while our method can distinguish the features of these two categories well. Therefore, for the categories of “Gaspes-Untrained” and “Vinyard-Untrained”, our method improves the classification accuracy by approximately 1.5–5.7% compared to CRSSNet and S3Net. On the UP dataset, CRSSNet and S3Net methods conflate the features belonging to the “Meadows” and “Bare soil” categories, failing to distinguish them well. However, in our method, the separability of these two categories is significantly better than CRSSNet and S3Net. Hence, for the categories of “Meadows” and “Bare soil”, our method improves the classification accuracy by approximately 6–16.4% compared to CRSSNet and S3Net.

The above experiments demonstrate that compared to the latest few-shot methods, our proposed method can extract features that are more conducive to classification, which also proves the advancement of the AGPS framework for few-shot HSI classification.

### 4.7. Ablation Analysis of the AGPS Framework

To verify the effectiveness of the advanced global prototypical representation learning strategy, ASPP-PA module, and FLC structure in the AGPS framework, we conducted an ablation study by removing each module from the AGPS framework.

Table 5 presents the results of the ablation study. The advanced global prototypical representation learning strategy with the addition of CL improves the OA on the three datasets by about 2–4%. The ASPP-PA module improves the OA on the three datasets by about 2–3%. The effect of the FLC on different datasets is also different. The FLC improves the OA of the model by about 25% on the UP dataset, while it only improves the accuracy by about 6% on the IP and SA datasets. This indicates that the pixels in the UP dataset rely more on the underlying detailed features during classification. Clearly, the experimental results show that all modules contribute to improving the model’s performance in terms of classification accuracy across the three datasets.

### 4.8. Analysis of the Impact of Different Numbers of Labeled Samples on the Performance of the AGPS Framework

We used GPN [24], DFSL + SVM, DFSL + NN, 3-D CNN, CRSSNet, and S3Net methods to evaluate the impact of varying numbers of labeled samples on the performance of the AGPS framework. GPN is a prototype network method that uses a patch-based global prototypical learning strategy. For each category, we randomly select {5, 10, 15, 20, 25} samples to train the network, and the remaining samples are used as unlabeled samples to evaluate the classification accuracy of the models.

In the IP dataset, since there are only 20 samples in the category “Oats”, we randomly select {5, 10, 15} labeled samples from each category. For the SA and UP datasets, we maintain the original settings.

**Figure 13:**
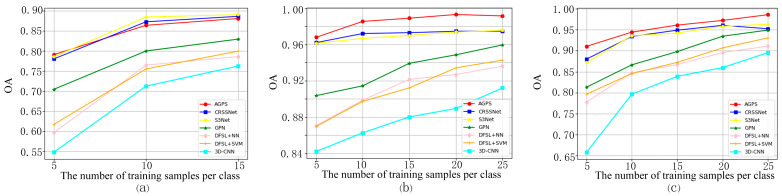
Evolution of OA as a function of number of training samples per class. (**a**) IP (**b**) SA (**c**) UP.

The OA evolution of the seven methods with different numbers of labeled samples on the three datasets is shown in Figure 13. For the IP dataset, the performance of the AGPS framework is comparable to CRSSNet and S3Net, and far better than the other four methods. For both the SA and UP datasets, our method outperforms all other methods consistently under varying numbers of labeled samples.

## 5. Conclusions

To further enhance the classification accuracy and prediction speed of few-shot HSI, this paper proposes an advanced global prototype segmentation (AGPS) framework. Within the AGPS framework, we propose SegNet, which takes the whole HSI as input and effectively avoids the disadvantages of patch-based methods. We embed the ASPP-PA module and the FLC structure into SegNet to enhance feature extraction capability. Finally, we combined CL with the global prototypical representation learning strategy to obtain more information from a few labeled samples to optimize our network. The experimental results demonstrate the superiority of the AGPS framework in terms of both classification accuracy and inference speed.

Although we have achieved good results, the method we proposed also has its limitations. Since our method takes the entire HSI as input, it leads to a high memory usage on the running device. In the future, we hope to overcome the high memory usage problem by exploring lightweight models and reducing HSI data redundancy.

## Figures and Tables

**Table 1:** Classification results (%) on the IP dataset.

No.	SVM	3D-CNN	FPGA	DFSL	DFSL + SVM	DFSL + NN	DCFSL	CMTL	S3Net	CRSSNet	Ours
1	72.20	95.12	97.83	92.68	96.75	96.75	87.80	95.12	**100.00**	**100.00**	**100.00**
2	34.27	37.70	49.23	39.85	36.38	38.65	45.40	51.09	**76.47**	75.32	58.98
3	39.18	19.77	42.65	56.85	38.34	42.79	57.21	**88.61**	57.93	62.39	56.13
4	50.34	32.51	72.99	71.55	77.16	68.10	84.91	80.17	99.44	**99.66**	98.92
5	69.75	88.45	68.08	64.64	73.92	71.20	78.45	65.90	**91.61**	89.41	77.67
6	66.36	73.65	68.90	89.52	86.25	76.18	88.97	**94.35**	93.48	90.97	80.17
7	89.13	81.82	**100.00**	**100.00**	97.10	**100.00**	**100.00**	**100.00**	**100.00**	**100.00**	**100.00**
8	68.73	53.35	94.97	73.36	81.82	74.84	73.57	58.99	98.75	97.93	**100.00**
9	86.87	**100.00**	**100.00**	**100.00**	75.56	**100.00**	**100.00**	**100.00**	**100.00**	**100.00**	**100.00**
10	37.49	41.35	75.61	51.09	52.22	47.98	65.25	76.32	65.64	58.13	**91.69**
11	33.96	66.71	68.46	52.28	59.96	57.95	59.14	58.98	67.01	68.99	**73.65**
12	31.43	37.40	54.63	29.08	36.56	38.21	43.37	71.97	64.52	60.82	**82.09**
13	86.50	85.71	99.02	98.00	98.00	97.50	99.00	**100.00**	91.50	90.30	99.90
14	62.93	62.57	90.11	82.30	84.63	83.44	90.16	81.75	**99.58**	99.01	91.13
15	28.08	56.42	95.33	54.07	74.10	62.23	62.47	87.93	92.76	95.33	**99.84**
16	90.91	90.36	**100.00**	**100.00**	**100.00**	**100.00**	**100.00**	98.86	99.32	99.20	**100.00**
OA%	45.85	54.76	68.82	59.55	61.69	59.65	66.40	71.35	78.60	78.03	**78.93**
AA%	59.24	63.93	79.86	72.20	73.05	72.24	77.23	81.88	87.37	86.72	**88.13**
Kappa%	39.68	48.72	64.71	54.43	56.78	54.55	63.10	67.97	75.98	75.33	**76.28**

**Table 2:** Classification results (%) on the SA data set.

Number	SVM	3D-CNN	FPGA	DFSL	DFSL + SVM	DFSL + NN	DCFSL	CMTL	S3Net	CRSSNet	Ours
1	97.57	95.29	99.90	94.66	73.92	95.63	99.55	99.25	**100.00**	**100.00**	**100.00**
2	87.43	97.20	89.77	99.00	96.85	99.09	99.71	99.52	**99.99**	99.97	**99.99**
3	82.95	91.45	**100.00**	72.35	96.28	94.01	93.68	99.95	99.99	**100.00**	99.85
4	99.11	97.31	99.35	93.38	99.11	99.54	99.45	87.40	**99.96**	99.83	96.04
5	94.29	91.24	95.59	83.84	80.72	90.58	90.39	94.09	95.04	95.57	**98.09**
6	98.36	98.80	97.62	99.37	91.63	98.47	99.27	**100.00**	99.67	99.55	96.15
7	94.39	99.69	99.97	98.68	97.73	99.81	99.04	99.52	**100.00**	**100.00**	**100.00**
8	59.99	66.40	49.64	68.26	82.33	77.74	72.61	85.76	88.77	88.91	**90.29**
9	96.09	96.25	99.78	97.42	94.44	91.13	99.74	99.77	**99.85**	99.71	99.30
10	71.45	70.72	74.64	76.54	80.96	60.98	84.51	95.69	93.20	**96.49**	91.23
11	91.25	93.15	**100.00**	98.02	93.38	95.99	98.17	99.53	99.57	99.45	**100.00**
12	97.22	99.65	95.38	98.49	97.94	93.13	99.04	96.20	97.65	97.97	**99.98**
13	97.30	92.63	**100.00**	92.97	95.79	**99.34**	98.97	95.39	91.13	93.12	87.70
14	91.84	93.56	98.69	98.78	98.87	98.06	97.99	99.72	98.22	98.21	**100.00**
15	60.52	68.02	95.04	81.34	91.13	77.54	74.12	69.89	96.66	94.24	**99.95**
16	81.45	81.41	96.45	88.12	90.57	85.05	90.62	**99.06**	97.00	98.06	98.39
OA%	80.71	84.20	85.88	86.22	86.95	87.05	88.53	91.73	96.13	96.20	**96.61**
AA%	87.58	89.56	93.25	90.10	90.08	91.01	93.55	95.05	97.29	**97.63**	97.31
Kappa%	78.61	82.46	84.41	84.74	85.51	85.63	87.27	90.79	95.70	95.73	**96.23**

**Table 3:** Classification results (%) on the UP dataset.

No.	SVM	3D-CNN	FPGA	DFSL	DFSL + SVM	DFSL + NN	DCFSL	CMTL	S3Net	CRSSNet	Ours
1	88.98	59.82	75.58	69.53	73.43	69.19	81.06	89.77	90.78	80.05	**91.56**
2	83.91	63.05	66.68	84.46	89.25	84.63	87.74	92.96	85.38	89.48	**95.57**
3	39.98	68.91	59.64	67.91	48.09	57.47	63.33	56.59	86.07	**88.79**	85.63
4	60.22	77.31	78.23	76.72	84.72	89.99	92.56	92.87	**96.38**	91.73	77.69
5	95.44	90.77	**100.00**	**100.00**	99.65	**100.00**	99.01	**100.00**	99.66	**100.00**	**100.00**
6	37.12	63.4	**95.66**	48.09	67.81	71.23	74.58	64.89	75.04	84.17	91.45
7	40.62	87.64	85.41	69.81	64.48	70.62	77.74	87.02	99.91	99.99	**100.00**
8	68.17	57.27	72.70	80.61	67.37	58.13	62.42	87.33	**87.23**	86.29	76.91
9	99.03	95.57	**99.37**	86.31	92.92	96.92	98.22	94.37	98.13	93.94	82.38
OA%	64.12	65.74	75.25	76.24	79.63	77.75	83.05	86.97	87.16	88.00	**91.08**
AA%	68.18	73.72	82.16	75.94	76.41	77.57	81.85	85.09	**90.95**	90.49	89.02
Kappa%	55.59	57.37	69.10	68.59	73.05	71.11	77.46	85.08	83.42	84.34	**88.21**

**Table 4:** The inference speed and computational cost of the models.

	S3Net	CRSSNet	DCFSL	AGPS
IP-Test time(s)	5.47	13.58	1.91	0.17
IP-GFLOPs	777.807	5790.635	438.894	16.235
UP-Test time(s)	17.11	32.92	7.19	0.63
UP- GFLOPs	1724.580	12,161.884	1810.940	139.328
SA-Test time(s)	29.21	68.01	10.53	0.79
SA- GFLOPs	4134.996	30,777.663	2334.755	72.740

**Table 5:** Ablation comparison of each variant of the AGPS framework.

	Baseline	NO FLC	NO ASPP-PA	NO CL	AGPS
IP (OA%)	65.23	72.97	76.87	74.26	78.93
UP (OA%)	62.68	64.08	88.24	89.16	91.08
SA (OA%)	85.84	89.99	93.45	93.75	96.61

## Data Availability

The datasets presented in this work are openly available on the website. Available online: https://www.ehu.eus/ccwintco/index.php/Hyperspectral_Remote_Sensing_Scenes (accessed on 5 June 2023).
